# Antimicrobial activity of organic acids against *Campylobacter* spp. and development of combinations—A synergistic effect?

**DOI:** 10.1371/journal.pone.0239312

**Published:** 2020-09-17

**Authors:** Elisa Peh, Sophie Kittler, Felix Reich, Corinna Kehrenberg

**Affiliations:** 1 Institute for Food Quality and Food Safety, University of Veterinary Medicine Hannover, Foundation, Hannover, Germany; 2 German Federal Institute for Risk Assessment (BfR), Berlin, Germany; 3 Institute for Veterinary Food Science, Justus-Liebig-University Giessen, Giessen, Germany; USDA-Agricultural Research Service, UNITED STATES

## Abstract

Contaminated poultry meat is considered to be the main source of human infection with *Campylobacter* spp., a pathogen that asymptomatically colonizes broiler chickens during fattening and contaminates carcasses during slaughter. To prevent or reduce the colonization of broiler flocks with *Campylobacter* spp., applying different organic acids, especially in combinations, via feed or drinking water seems to be a promising approach. However, only very few combinations of organic acids have been tested for their antibacterial efficacy against *Campylobacter* spp. Therefore, the *in vitro* susceptibility of 30 *Campylobacter* spp. isolates (20 *C*. *jejuni* and ten *C*. *coli*) to ten organic acids and ten combinations was determined. The testing of minimum inhibitory concentration (MIC) values was performed at pH 6.0 and 7.3 by using the broth microdilution method and included the following organic acids: Caprylic acid, sorbic acid, caproic acid, benzoic acid, ascorbic acid, propionic acid, acetic acid, formic acid, fumaric acid and tartaric acid and combinations thereof. The lowest MIC values were seen for caprylic acid (MIC range at pH 7.3: 0.5–2 mmol/L) and sorbic acid (MIC range at pH 7.3: 1–4 mmol/L). One to two dilution steps lower MIC values were determined at the lower pH value of 6.0. Furthermore, ten combinations consisting of three to five organic acids were developed. In addition to the tested antibacterial activity, other criteria were included such as approval as feed additives, reported synergistic effects and chemical properties. For nine of ten combinations, the MIC_90_ values of the organic acids decreased 1.25- to 241.5-fold compared to the MIC_90_ values for the individual substances. Furthermore, nine of ten combinations exhibited synergistic activities against two or more of the tested *C*. *jejuni* and *C*. *coli* isolates. A combination of caprylic acid, sorbic acid and caproic acid exhibited synergistic activities against the largest number of *Campylobacter* spp. isolates (six *C*. *jejuni* and four *C*. *coli*) with fractional inhibitory concentration (FIC) indices (∑FIC) ranging from 0.33 to 1.42. This study shows *in vitro* synergistic activities of different organic acids in combinations against the major *Campylobacter* species and could therefore be a promising basis for reducing *Campylobacter* spp. *in vivo*.

## Introduction

Campylobacteriosis is one of the leading foodborne gastrointestinal diseases worldwide [1] and the most frequently reported zoonosis in the European Union with more than 246,000 reported cases in 2018 [2]. *Campylobacter* (*C*.) *jejuni* and *C*. *coli* are the species that most frequently cause gastrointestinal diseases [3]. Clinical manifestations of human infection with *Campylobacter* spp. include acute aqueous or bloody diarrhea, fever and occasionally severe sequelae such as the Guillain-Barré syndrome and Miller Fisher syndrome [1]. It is assumed that contaminated poultry meat is the major source for human infection with *Campylobacter* spp. [4]. Contamination of carcasses is mainly caused by faecal contamination during the slaughter process and currently a large number of slaughter batches are affected [5]. Comprehensive strategies for reducing microbial contamination of carcasses and controlling *Campylobacter* infections are urgently needed. Reducing *Campylobacter* spp. in primary production is considered to be most effective for minimizing human infections [4]. Different *in vivo* studies demonstrated the potential of organic acids to decrease the susceptibility for colonization or to reduce caecal concentrations of *Campylobacter* spp. when applicated via feed or water [6–9] However, as pointed out in the scientific opinion published by the European Food Safety Authority [4], results of previous *in vivo* experiments concerning the effectiveness of organic acids were inconsistent, indicating that further research using standardized methods is urgently needed.

A couple of *in vitro* studies have addressed the problem and investigated the antibacterial effect of organic acids against *Campylobacter* spp. However, it is difficult to compare the results because different methods and techniques were used to determine the susceptibility of the isolates. For example, Molatová et al. [10] performed susceptibility tests with only one *C*. *jejuni* strain using a SYBR Green-based real-time PCR, while Grilli et al. [11] determined MIC values of three *C*. *jejuni* isolates by using the broth macrodilution method. Two other studies performed susceptibility tests by using the broth microdilution method, but MIC values were determined either at pH 6 or 7.5 [12] or without any adjustment of the pH [13]. Furthermore, despite promising results of studies using combinations of organic acids *in vivo* [8, 14, 15], very few *in vitro* studies investigated the susceptibility status of *Campylobacter* spp. isolates to combinations of organic acids. Additionally, these studies did not always provide complete information on the composition and the selection of substances for the combinations.

Therefore, this study aimed to evaluate the antibacterial effect of a variety of organic acids *in vitro*. Minimum inhibitory concentration (MIC) values of ten organic acids were determined individually and in various combinations against current *C*. *jejuni* and *C*. *coli* field isolates. Subsequently, we investigated the interactions between the compounds based on the fractional inhibitory concentration (FIC) index.

## Materials and methods

### Bacterial strains

A total of 20 *C*. *jejuni* and ten *C*. *coli* isolates were included in this study. The strain collection included type strains *C*. *jejuni* DSM 4688 and *C*. *coli* DSM 4689 (German Collection of Microorganisms and Cell Cultures, Leibnitz-Institute, Braunschweig, Germany), whole genome-sequenced strain *C*. *jejuni* BfR-CA-14430, *C*. *coli* strain BfR-CA-09557 and *C*. *jejuni* strain ATCC 81–176 (American Type Culture Collection (ATCC), Manassas, VA, USA). Additionally, 17 *C*. *jejuni* and eight *C*. *coli* field isolates of avian origin, representing part of the strain collection of the Institute of Food Quality and Food Safety, University of Veterinary Medicine Hannover, Hannover, Germany and the Institute for Veterinary Food Science, Giessen, Germany were used for susceptibility testing. The field isolates were collected between July 2005 and January 2018 on the basis of epidemiological unrelatedness. Species confirmation of all isolates was carried out by MALDI-TOF mass spectrometry (Bruker Daltoniks GmbH, Bremen, Germany). All isolates were stored in cryotubes (Carl Roth GmbH + Co. KG, Karlsruhe, Germany) at -80 °C. Prior to use, isolates were plated out on Columbia agar supplemented with sheep blood (Oxoid Deutschland GmbH, Wesel, Germany) and incubated for 48 hours at 42 ± 1 °C under microaerobic conditions (10% CO_2_, 5% O_2_, and 85% N_2_).

### Organic acids and test ranges

Ten organic acids were tested for their antibacterial effect: formic acid, propionic acid, ascorbic acid, tartaric acid, sorbic acid, benzoic acid, fumaric acid, caprylic acid, caproic acid (Carl Roth GmbH + Co. KG, Karlsruhe, Germany) and acetic acid (E. Merck KG, Darmstadt, Germany). Since it is considered that the antimicrobial efficacy of organic acids is related to the pH value [16], susceptibility tests were performed at two different pH values. This took into account that the dissociation state plays an important role for their effectiveness and that an application in poultry primary production, e.g. via drinking water, is expected to cause a pH shift with acidification of the water. For better comparability with results of previous studies [11, 13, 17], the concentrations of organic acids are given in mmol/L. The stock solutions of organic acids were prepared at double strength of the respective first dilution level in cation adjusted Mueller-Hinton broth (CAMH, Carl Roth GmbH + Co. KG, Karlsruhe, Germany), and adjusted to pH 6.0 or pH 7.3 using 2 mol/L and 8 mol/L sodium hydroxide. Subsequently, two-fold serial dilution series were prepared in CAMH broth previously adjusted to pH 6.0 or pH 7.3. The following final concentrations (which are specified after inoculation) and ranges were included: 0.5–512 mmol/L (formic acid, acetic acid, propionic acid, tartaric acid, fumaric acid), 0.031–32 mmol/L (ascorbic acid, caprylic acid), and 0.063–64 mmol/L (benzoic acid, sorbic acid, caproic acid). A volume of 50 μL of each dilution was then added to the wells of a microtiter plate (Sarstedt AG & Co. KG, Nümbrecht, Germany).

### Determining the susceptibility of *Campylobacter* isolates towards organic acids

For determining minimal inhibitory concentration (MIC) values of organic acids, the broth microdilution method was used. Procedures regarding inoculum density, growth medium, incubation time and conditions were performed in accordance with the recommendations given in the Clinical and Laboratory Standards Institute (CLSI) document VET01-A4 [18]. The tests were performed in U-shaped bottom 96-well microtiter plates (Sarstedt AG & Co. KG, Nümbrecht, Germany). Colonies from overnight cultures were suspended in sodium chloride (0.85%) and adjusted to a turbidity in accordance with McFarland standard 0.5. The suspension was diluted 1:100 in CAMH broth and adjusted to pH 6.0 or pH 7.3, respectively. A volume of 50 μL of this suspension was added into the wells of the microtiter plate containing 50 μL of the double concentrated organic acid to achieve a final bacterial concentration of 5 x 10^5^ CFU/mL. The microtiter plates were incubated for 48 h at 42 ± 1 °C under microaerobic conditions. *C*. *jejuni* strain DSM 4688 and *C*. *coli* strain DSM 4689 served as quality control strains and were included in every batch of MIC determinations. The MICs of the two quality control strains were determined in advance in three independent experiments by using the broth microdilution method as well as the broth macrodilution method, similar to a previous study on biocide testing (Rensch et al. 2013).

### Development of combinations of organic acids

In accordance with the following criteria, ten combinations of organic acids termed CA to CJ were chosen, each consisting of three to five components. The respective combinations comprised the following organic acids: CA (caprylic acid, sorbic acid, caproic acid), CB (caprylic acid, sorbic acid, caproic acid, ascorbic acid), CC (caprylic acid, sorbic acid, caproic acid, benzoic acid), CD (caprylic acid, sorbic acid, caproic acid, ascorbic acid, benzoic acid), CE (caprylic acid, sorbic acid, caproic acid, benzoic acid, propionic acid), CF (sorbic acid, ascorbic acid, benzoic acid), CG (sorbic acid, ascorbic acid, benzoic acid, propionic acid), CH (sorbic acid, benzoic acid, propionic acid), CI (sorbic acid, benzoic acid, propionic acid, acetic acid), CJ (sorbic acid, benzoic acid, propionic acid, acetic acid, formic acid). The ratios of organic acids were 3:2:1 for three components, 4:3:2:1 for four components, and 5:4:3:2:1 for five components (Table 1).

**Table 1:** Composition of the ten tested blends of organic acids based on the dilution level 64 mmol/L.

	Formulation of the combinations (in mmol/L)^a^							
Combination	Caprylic acid	Sorbic acid	Caproic acid	Ascorbic acid	Benzoic acid	Propionic acid	Acetic acid	Formic acid
CA	32.0	21.3	10.7					
CB	25.6	19.2	12.8	6.4				
CC	25.6	19.2	12.8		6.4			
CD	21.3	17.1	12.8	8.5	4.3			
CE	21.3	17.1	12.8		8.5	4.3		
CF		32.0		21.3	10.7			
CG		25.6		19.2	12.8	6.4		
CH		32.0			21.3	10.7		
CI		25.6			19.2	12.8	6.4	
CJ		21.3			17.1	12.8	8.5	4.3

^a^Combinations consisted of three to five organic acids with the following ratios: 3:2:1 (three components, red areas), 4:3:2:1 (four components, blue areas), 5:4:3:2:1 (five components, green areas).

Since in previous studies, synergistic effects were reported for short chain fatty acids in combination with both phenolic compounds and medium chain fatty acids [17, 19], all combinations at least included either caprylic acid as a medium chain fatty acid or benzoic acid as a phenolic acid, or combinations thereof in order to use these reported synergistic effects. The mixtures of organic acids consisted either exclusively of organic acids listed as authorized feed additives in the European Union (combinations CF, CG, CH, CI, CJ) or contained caprylic acid and caproic acid as substances yet to be approved (combinations CA, CB, CC, CD, CE) [20]. The organic acids were selected on the basis of the results of the testing of individual substances (as they showed very low MIC values). The separate grouping of approved versus non-approved acids was done under the consideration of regulatory and practical aspects, since non-approved acids might be effective but not directly applicable. Combinations were prepared with respect to the chemical structure and octanol/water partition coefficient as a measure of hydrophobicity (Table 2) to evaluate possible synergistic effects between organic acids with different chemical properties. In that respect, ascorbic acid was included in this study as the only vinylogous carboxylic acid with the lowest octanol/water partition coefficient of all tested organic acids being included in four combinations (CB, CD, CF, CG).

**Table 2:** List of chemical properties of the organic acids.

	Octanol/water partitition coefficient	Number of carbon atoms	Number of carbon-carbon double bonds	Number of carboxy groups	Dissociation constant (pK_a_1)^d^
Caprylic^a^	3.05	8	0	1	4.89
Sorbic	1.33	6	2	1	4.76
Caproic^b^	1.92	6	0	1	4.88
Ascorbic	-1.85	6	1	0	4.70
Benzoic^c^	1.87	7	3	1	4.19
Propionic^b^	0.33	3	0	1	4.88
Acetic^b^	-0.17	2	0	1	4.76
Formic^b^	-0.54	1	0	1	3.75
Tartaric	-1.35	4	0	2	3.40
Fumaric	0.46	4	1	2	3.03

^a^ Medium chain fatty acid (MCFA).

^b^ Short chain fatty acid (SCFA).

^c^ Phenolic acid.

^d^ pK_a_1 = first (lowest) pK_a_.

The antibacterial effectiveness of the organic acids determined their selection and the proportion of organic acids in the mixtures (Table 1). The ratios of the combinations were 3:2:1 for three components, 4:3:2:1 for four components and 5:4:3:2:1 for five components. In all combinations, the organic acid with the lowest MIC_90_ value (lowest concentration of the organic acid at which 90% of the bacteria were inhibited or killed) constituted the largest proportion, followed by organic acids with the next lowest MIC_90_ values, respectively. Due to their low effectiveness based on their high MIC_50_ and MIC_90_ values compared to the other substances, the dicarboxylic acids tartaric acid and fumaric acid were not included in the mixtures.

For each combination of organic acids, stock solutions were prepared in CAMH broth with a total organic acid concentration of 64 mmol/L according to Table 1. The stock solutions were adjusted to pH 7.3 using 2 mol/L and 8 mol/L sodium hydroxide and 11 serial two-fold dilutions were prepared in CAMH broth.

### Susceptibility testing of combinations of organic acids

To determine the minimum inhibitory concentrations of the combinations of organic acids, broth microdilution assays were performed as described above. MIC_90_ values of the combinations of organic acids were calculated.

Then, the fractional inhibitory concentration (FIC) index was calculated for each combination of organic acids to evaluate possible synergistic activities [21, 22]. A synergistic effect refers to a combination of two or more components that causes a greater effect than the sum of their individual effects [23].

First, MICs of the respective organic acids in each combination were transformed into fractional inhibitory concentrations (FICs) as follows [21]:

$${\text{FIC}}_{\text{organic}\;\text{acid}}=\frac{{\text{MIC}}_{\text{organic}\;\text{acid}}\;\text{in}\;\text{combination}}{{\text{MIC}}_{\text{organic}\;\text{acid}}\;\text{alone}}$$

Second, the fractional inhibitory concentration (FIC) index (∑FIC) was calculated using the standard formula as described earlier [21, 22]:

$$\sum_{k=1}^{n}\text{FIC}$$

Synergistic activities were defined as ∑FIC ≤ 0.5, indifference was defined as ∑FIC > 0.5 to < 2, and antagonism was defined as ∑FIC ≥ 2 [21].

## Results

### Antimicrobial susceptibility testing of individual organic acids

The distribution of MIC values determined in the testing of single organic acids is shown in Table 3A and 3B. The tests were performed using 20 *C*. *jejuni* and ten *C*. *coli* isolates; therefore, the results were presented separately by species. The MIC_50_ and MIC_90_ values were defined as the lowest concentration of organic acids at which 50% and 90% of the isolates were inhibited, respectively. The MIC_50_ and MIC_90_ values were 1- to 3-fold lower at pH 6.0 than at pH 7.3, except for fumaric acid with identical MIC_50_ and MIC_90_ values of 256 mmol/L determined for both pH conditions and both bacterial species. The overall distribution of the MIC values for the *C*. *jejuni* and *C*. *coli* isolates was quite similar (Table 3A and 3B). A comparison of the susceptibility of both bacterial species based on the MIC_90_ values showed a similar ranking of organic acids. The only difference was seen for benzoic acid and ascorbic acid. Regarding these substances, *C*. *jejuni*, showed lower MICs for benzoic acid (MIC_90_ of 8 mmol/L at pH 7.3) than for ascorbic acid (MIC_90_ of 16 mmol/L at pH 7.3). In contrast, the tested *C*. *coli* isolates revealed lower MIC_90_ values for ascorbic acid at pH 7.3 (8 mmol/L) than for benzoic acid (16 mmol/L).

**Table 3:** Distribution of minimum inhibitory concentrations (MIC) values of 20 *C*. *jejuni* and ten *C*. *coli* isolates for ten organic acids at pH 6.0 and 7.3 using the broth microdilution method.

			Number of isolates with MIC (mmol/L) value of																
Organic acid	Species	pH	0.03	0.06	0.13	0.25	0.5	1	2	4	8	16	32	64	128	256	512	MIC_50_	MIC_90_
Caprylic acid	*C*. *jejuni*	6.0	0	0	0	1	11	8	0	0	0	0	0					0.5	1
		7.3	0	0	0	0	0	4	16	0	0	0	0					2	2
	*C*. *coli*	6.0	0	0	0	2	6	2	0	0	0	0	0					0.5	1
		7.3	0	0	0	0	1	3	6	0	0	0	0					2	2
Sorbic acid	*C*. *jejuni*	6.0		0	0	0	15	5	0	0	0	0	0	0				0.5	1
		7.3		0	0	0	0	2	15	3	0	0	0	0				2	4
	*C*. *coli*	6.0		0	0	2	5	3	0	0	0	0	0	0				0.5	1
		7.3		0	0	0	0	3	5	2	0	0	0	0				2	4
Caproic acid	*C*. *jejuni*	6.0		0	0	0	6	10	3	1	0	0	0	0				1	2
		7.3		0	0	0	0	0	4	15	1	0	0	0				4	4
	*C*. *coli*	6.0		0	0	0	5	3	2	0	0	0	0	0				0.5	2
		7.3		0	0	0	0	0	2	6	2	0	0	0				4	8
Ascorbic acid	*C*. *jejuni*	6.0	0	0	0	0	2	7	9	2	0	0	0					2	2
		7.3	0	0	0	0	0	0	3	6	8	3	0					8	16
	*C*. *coli*	6.0	0	0	0	0	1	3	5	1	0	0	0					2	2
		7.3	0	0	0	0	0	0	0	5	4	1	0					4	8
Benzoic acid	*C*. *jejuni*	6.0		0	0	0	0	2	16	2	0	0	0	0				2	2
		7.3		0	0	0	0	0	0	1	17	2	0	0				8	8
	*C*. *coli*	6.0		0	0	0	0	0	5	4	1	0	0	0				2	4
		7.3		0	0	0	0	0	0	0	4	6	0	0				16	16
Propionic acid	*C*. *jejuni*	6.0					0	0	4	10	4	2	0	0	0	0	0	4	8
		7.3					0	0	0	0	5	7	6	2	0	0	0	16	32
	*C*. *coli*	6.0					0	0	0	2	4	4	0	0	0	0	0	8	16
		7.3					0	0	0	0	0	3	5	2	0	0	0	32	64
Acetic acid	*C*. *jejuni*	6.0					0	0	1	4	9	5	1	0	0	0	0	8	16
		7.3					0	0	0	0	0	4	10	4	2	0	0	32	64
	*C*. *coli*	6.0					0	0	1	2	4	3	0	0	0	0	0	8	16
		7.3					0	0	0	0	0	2	6	1	1	0	0	32	64
Formic acid	*C*. *jejuni*	6.0					0	0	0	2	7	9	2	0	0	0	0	16	16
		7.3					0	0	0	0	0	0	3	13	4	0	0	64	128
	*C*. *coli*	6.0					0	0	0	1	4	4	1	0	0	0	0	8	16
		7.3					0	0	0	0	0	0	6	3	1	0	0	32	64
Tartaric acid	*C*. *jejuni*	6.0					0	0	0	0	2	3	6	4	5	0	0	32	128
		7.3					0	0	0	0	0	2	2	4	7	5	0	128	256
	*C*. *coli*	6.0					0	0	0	0	5	0	4	1	0	0	0	8	32
		7.3					0	0	0	0	0	4	1	2	2	1	0	32	128
Fumaric acid	*C*. *jejuni*	6.0					0	0	0	0	0	0	0	1	13	6	0	128	256
		7.3					0	0	0	0	0	0	0	0	2	18	0	256	256
	*C*. *coli*	6.0					0	0	0	0	0	0	0	0	7	3	0	128	256
		7.3					0	0	0	0	0	0	0	0	3	7	0	256	256

The white areas represent the tested range of organic acids.

The lowest MIC values were detected for caprylic acid, followed by sorbic acid. For both organic acids, *C*. *jejuni* and *C*. *coli* isolates yielded MIC_50_ values of 0.5 mmol/L at pH 6.0, 2 mmol/l at pH 7.3 and MIC_90_ values of 1 mmol/L at pH 6.0. At pH 7.3, caprylic acid revealed MIC_90_ values (2 mmol/L) half of these of sorbic acid (4 mmol/L) against *C*. *jejuni* and *C*. *coli* isolates. The highest MIC values were observed for fumaric acid (MIC_50_ values: 128 mmol/L at pH 6.0, 256 mmol/L at pH 7.3; MIC_90_ values: 256 mmol/L at pH 6.0, 256 mmol/L at pH 7.3 for *C*. *jejuni* and *C*. *coli* isolates) and tartaric acid with MIC_90_ values of 128 mmol/L at pH 6.0 and 256 mmol/L at pH 7.3 for the two species.

### Antimicrobial susceptibility testing of combinations of organic acids

The MIC_90_ values of the combined organic acids are presented in Table 4. According to Grilli et al. [11], the MIC_90_ values of the organic acids in combination were calculated using the MIC_90_ values of the combination as a whole and the respective proportions of the individual components. Combining organic acids decreased the MIC_90_ values of each organic acid except sorbic acid in combination with ascorbic acid or benzoic acid (CF combination) when testing *C*. *coli*, with MIC_90_ values of 4 mmol/L achieved both alone and in combination. However, looking at the results for *C*. *jejuni* isolates, the MIC_90_ value of sorbic acid in the CF combination was reduced by a factor of 2. Due to their low MIC_90_ values, either caprylic acid (used in CA–CE combinations) or sorbic acid (CF–CJ combinations) had the largest proportion in the mixtures and, thus, showed the lowest reductions in MIC_90_ values compared to single testing. The MIC_90_ values of caprylic acid (CA–CE combinations) decreased only 1.25- to 2.5-fold for both bacterial species. Similarly, the CG, CH, CI and CJ combinations resulted in 1.5- to 2.5-fold reductions in the MIC_90_ values of sorbic acid for *C*. *jejuni* and *C*. *coli* isolates. The highest reduction in an MIC_90_ value was observed for formic acid in the CJ combination, showing a 241.5-fold decrease in the MIC_90_ value for *C*. *jejuni* isolates.

**Table 4:** MIC_90_ values of eight organic acids tested alone and in combinations (CA-CJ) against 20 *C*. *jejuni* (A) and ten *C*. *coli* (B) isolates determined by the broth microdilution method at pH 7.3.

(A)									
		**caprylic acid**	**sorbic acid**	**caproic acid**	**ascorbic acid**	**benzoic acid**	**propionic acid**	**acetic acid**	**formic acid**
**MIC**_**90**_ **(mmol/L) alone**^a^		2.00	4.00	4.00	16.00	8.00	32.00	64.00	128.00
**MIC**_**90**_ **(mmol/L) in combination**^a^^,^^b^	**CA**	1.00	0.67	0.33					
	**CB**	1.60	1.20	0.80	0.40				
	**CC**	0.80	0.60	0.40		0.20			
	**CD**	1.33	1.07	0.80	0.53	0.27			
	**CE**	1.33	1.07	0.80		0.53	0.27		
	**CF**		2.00		1.33	0.67			
	**CG**		1.60		1.20	0.80	0.40		
	**CH**		2.00			1.33	0.67		
	**CI**		1.60			1.20	0.80	0.40	
	**CJ**		2.67			2.13	1.60	1.07	0.53
(B)									
		**caprylic acid**	**sorbic acid**	**caproic acid**	**ascorbic acid**	**benzoic acid**	**propionic acid**	**acetic acid**	**formic acid**
**MIC**_**90**_ **(mmol/L) alone**^a^		2.00	4.00	8.00	8.00	16.00	64.00	64.00	64.00
**MIC**_**90**_ **(mmol/l) in combination**^a^^,^^b^	**CA**	1.00	0.67	0.33					
	**CB**	1.60	1.20	0.80	0.40				
	**CC**	0.80	0.60	0.40		0.20			
	**CD**	1.33	1.07	0.80	0.53	0.27			
	**CE**	1.33	1.07	0.80		0.53	0.27		
	**CF**		4.00		2.67	1.33			
	**CG**		1.60		1.20	0.80	0.40		
	**CH**		2.00			1.33	0.67		
	**CI**		1.60			1.20	0.80	0.40	
	**CJ**		2.67			2.13	1.60	1.07	0.53

^a^Green areas indicate lower MIC_90_ values, while yellow and red areas indicate higher MIC values.

^b^MIC_90_ values of the organic acids in combination were calculated according to the MIC_90_ values of the combination as a total and the respective proportion of the individual components.

### Fractional inhibitory concentration index

The ∑FIC of the organic acids in combination calculated after testing of *C*. *jejuni* and *C*. *coli* isolates is presented in Table 5. The results indicated synergistic or indifferent interactions, while no antagonistic interactions were observed. The ∑FIC ranged from 0.28 to 2.75 for all combinations of organic acids and isolates. The highest number of synergistic effects against the tested isolates was observed for the combination CA consisting of caprylic acid, sorbic acid and caproic acid. Results showed synergistic activities against six *C*. *jejuni* and four *C*. *coli* isolates with ∑FIC ranging from 0.33 to 1.42 for *C*. *jejuni* and from 0.34 to 1.42 for *C*. *coli*. The combination CI consisting of sorbic acid, benzoic acid, propionic acid and acetic acid showed synergism against five *C*. *jejuni* and four *C*. *coli* isolates, presenting ∑FIC ranges of 0.46 to 1.81 for *C*. *jejuni* and 0.46 to 1.79 *for C*. *coli*. The CD combination consisting of caprylic acid, sorbic acid, caproic acid, ascorbic acid and benzoic acid exhibited exclusively indifferent interactions against all tested isolates (Table 5).

**Table 5:** Results of testing combined organic acids for synergistic activity using 20 *C*. *jejuni* and ten *C*. *coli* isolates.

	*C*. *jejuni* (n = 20)		*C*. *coli* (n = 10)	
Combination^c^	Number of isolates exhibiting synergy^b^	∑FIC range^a^	Number of isolates exhibiting synergy	∑FIC range
CA	6	0.33–1.42	4	0.34–1.42
CB	2	0.41–2.60	0	0.61–2.50
CC	3	0.41–1.65	2	0.41–1.23
CD	0	0.60–2.37	0	0.75–2.28
CE	3	0.36–1.22	0	0.54–2.11
CF	2	0.33–1.75	0	0.63–2.75
CG	2	0.30–1.55	0	0.51–2.01
CH	2	0.28–2.21	1	0.28–1.19
CI	5	0.46–1.81	4	0.46–1.79
CJ	6	0.39–1.68	2	0.39–1.57

^a^The fractional inhibitory concentration (FIC) index was calculated as the sum of the FICs for each individual organic acid.

^b^∑FIC ≤ 0.5 was defined as synergism and ∑FIC >0.5 to <2 was defined as indifference.

^c^Combinations CA-CJ consisted of three to five organic acids with the following compositions: CA (caprylic acid, sorbic acid, caproic acid), CB (caprylic acid, sorbic acid, caproic acid, ascorbic acid), CC (caprylic acid, sorbic acid, caproic acid, benzoic acid), CD (caprylic acid, sorbic acid, caproic acid, ascorbic acid, benzoic acid), CE (caprylic acid, sorbic acid, caproic acid, benzoic acid, propionic acid), CF (sorbic acid, ascorbic acid, benzoic acid), CG (sorbic acid, ascorbic acid, benzoic acid, propionic acid), CH (sorbic acid, benzoic acid, propionic acid), CI (sorbic acid, benzoic acid, propionic acid, acetic acid), CJ (sorbic acid, benzoic acid, propionic acid, acetic acid, formic acid).

## Discussion

In this study, the antibacterial activities of organic acids both alone and in different combinations were determined by using the broth microdilution method.

The MIC values of several organic acids differed in part strongly to those determined for two *C*. *jejuni* strains in a previous study [11]. Compared to the results of the present study, the authors found higher MIC values for caprylic acid (62.5 mmol/L), sorbic acid (500 mmol/L), acetic acid (>1000 mmol/L), formic acid (>1000 mmol/L), fumaric acid (>1000 mmol/L) and tartaric acid (>1000 mmol/L) [11]. However, MIC values of propionic acid (62.5 mml/L) and benzoic acid (31.25 mmol/L) differed only slightly from our results. Most likely, the partly varying results were due to differences regarding the evaluation of the MIC values. According to the CLSI standards for antimicrobial susceptibility tests for bacteria isolated from animals, the MIC value is defined as the lowest concentration that inhibits visible growth. Deviating from this, Grilli et al. [11] defined the MIC value as the lowest concentration effective in killing more than 99.9% of the initial inoculum as determined by a colony-count technique. As a consequence, the observed concentrations were most likely higher for organic acids that are rather bacteriostatic than bactericidal. In addition, different methods and techniques were used for susceptibility testing. Unlike our study, the MIC values were determined by the broth macrodilution method at pH 6.5 and by using Brain Heart Infusion broth as a test medium [11], which could lead to different results. Compared to our study, more similar results were obtained by two other studies that defined the MIC values in accordance with the CLSI standards and performed susceptibility tests using the broth microdilution method [12, 13]. Consistent with our results, Hermans et al. [12] observed MIC values for caprylic acid and caproic acid ranging between 2 and 4 mmol/L at pH 7.3. Beier et al. [13] reported MIC_90_ values similar to our results for propionic acid (13.82 mmol/L), formic acid (44.5 mmol/L) and acetic acid (34.1 mmol/L), although the pH values had not been previously adjusted. Thus, they performed susceptibility tests at widely varying pH values depending on the concentration and the pK_a_ value of the respective organic acid.

As expected, the present study demonstrated that the pH value affects the antibacterial activity of organic acids, as all organic acids yielded lower MIC values at pH 6.0 compared to pH 7.3. The widely assumed reason for this is that organic acids are only able to cross the cell membrane in an undissociated form [24]. The proportion of those in an undissociated form depends on the pK_a_ value in combination with the external pH value of the medium [11]. In the bacterial cell, the higher pH value leads to a dissociation of the organic acids into their anions and protons. Cytoplasmic acidification caused by protons lead to disruption of certain cell functions [16]. Additionally, accumulation of anions in the cytoplasm has been proposed to disrupt metabolic functions and to cause increased osmotic pressure and cell death [25]. However, the enhancing effect of acidification on the antibacterial activity of organic acids might be limited to drinking water and feed themselves, as the pH value was observed to increase in the intestines due to the buffering effect of the intestinal contents [26].

In the present study, synergistic activities were shown for the organic acids in all combinations except for the CD combination. For example, the CJ combination consisting of sorbic acid, benzoic acid, propionic acid, acetic acid and formic acid exhibited synergistic interactions against six *C*. *jejuni* and two *C*. *coli* isolates. For three of these organic acids, a previous study showed strong synergistic activities against *Campylobacter* spp. *in vitro* when added to a mixture of water and broiler feed [27]. At pH 4.5, combinations of formic acid, acetic acid and propionic acid resulted in higher reduction rates of *Campylobacter* spp. than the individual organic acids [27]. Furthermore, in an *in vivo* study conducted by Skånseng et al. [8], neither the addition of formic acid nor potassium sorbate to broiler feed did lead to reduced contamination levels, whereas the application of a combination of 2.0% formic acid and 0.1% potassium sorbate prevented *C*. *jejuni* colonization in chickens. Kim and Rhee [17] observed synergistic activities of three medium chain fatty acids (MCFAs) including caprylic acid and four different short chain fatty acids (SCFAs) against *E*. *coli* as indicated by higher rates of bacterial reduction compared to the individual treatment. In this study, four of five combinations consisting of caprylic acid as a MCFA and different SCFA (combinations CA, CB, CC, CE) exhibited synergistic interactions against two or more *Campylobacter* spp. isolates. The underlying mechanisms for the reported synergistic activities between different organic acids are still unclear. Synergism can occur when organic acids with mutually reinforcing modes of actions are used in combination. In fact, the bacterial cell membrane was found to be a target for antibacterial action of several MCFA [17, 28, 29] and phenolic acids [30]. In contrast, SCFA exhibited antibacterial activities without causing damage to the cell wall [17, 27]. Accordingly, Kim and Rhee [17] proposed that MCFA are likely to damage the bacterial cell membrane and thus may accelerate the influx of SCFA.

The combination of organic acids allowed a reduction in the concentrations of nearly all components required for effective antimicrobial activity as shown in the reduced MIC_90_ values. This finding offers an important advantage. Several authors observed reduced feed consumption when organic acids exceeding a certain concentration were added to feed or water [31–33]. It was suggested that the strong taste of organic acids might decrease palatability thereof [34]. Thus, concentrations of single organic acids required for effective antibacterial activity might exceed the level of acceptance in broilers if used individually. For example, propionic acid was observed to decrease the feed intake and weight gain of broilers when added to drinking water at a concentration of 90 mmol/L [35] which is only slightly higher than the MIC_90_ value determined for propionic acid against *C*. *coli* isolates (64 mmol/L). Considering the dilution effect of intestinal contents, acid concentrations far higher than those of the MIC values would be required for effective antimicrobial activity. This would most probably lead to adverse effects on broiler performance due to reduced intake of water or feed. Such a problem could be overcome by treatment with optimized combinations that reach antimicrobial efficacy, while the concentration of the individual components is sufficiently low for sensory acceptance, especially if they have synergistic activities. However, it should be noted that the concentrations of organic acids in the entire gastrointestinal tract have decreased due to absorption and metabolism processes [36]. As suggested by various authors, this can be counteracted by the use of coated organic acids [11, 12]. Accordingly, it was observed that encapsulation of MCFAs increased the efficiency in reducing *C*. *jejuni* counts in faecal samples [37]. Thus, with regard to future *in vivo* studies with optimized combinations, it might therefore be worth to consider the administration of organic acids in microencapsulated form.

In conclusion, the results of the present study demonstrated the high potential of combining organic acids against *Campylobacter* spp. *in vitro* using a systematic stratified approach for selection. Synergistic activities were proven for nine of ten combinations of organic acids, while combining different organic acids at least decreased the MIC_90_ values of nearly all individual compounds. This study provides a database of effective combinations of organic acids against *Campylobacter* spp. evaluated *in vitro* by a highly standardized method. Further research using animal models is necessary to verify the antibacterial efficacy of the combined organic acids *in vivo* when applied via feed or drinking water.

## Supporting information

S1 Table The fractional inhibitory concentration indices (∑FIC) of ten combinations of organic acids tested against 20 *C*. *jejuni* and ten *C*. *coli* isolates. (DOCX)
