# Supplementary material for: Antimicrobial activity of organic acids against Campylobacter spp. and development of combinations—A synergistic effect?
Source: PLoS One. 2020 Sep 17;15(9):e0239312. doi: 10.1371/journal.pone.0239312 (PMC7497993; doi:10.1371/journal.pone.0239312)
Supplement: S1 Table — (DOCX) [file pone.0239312.s001.docx]

**S1 Table. The fractional inhibitory concentration indices (∑FIC) of ten combinations of organic acids tested against 20 *C. jejuni* and ten *C. coli* isolates.**

The grey areas represent combinations of organic acids exhibiting synergistic interactions (∑FIC ≤ 0.5) against the respective isolate.

|  | **Combination** | | | | | | | | | |
| --- | --- | --- | --- | --- | --- | --- | --- | --- | --- | --- |
|  | CA | CB | CC | CD | CE | CF | CG | CH | CI | CJ |
| ***Campylobacter jejuni*** | ∑FIC | ∑FIC | ∑FIC | ∑FIC | ∑FIC | ∑FIC | ∑FIC | ∑FIC | ∑FIC | ∑FIC |
| Campy_If37 | 0.46 | 0.83 | 0.83 | 0.75 | 0.74 | 1.25 | 0.54 | 0.60 | 0.51 | 0.89 |
| Campy_If70 | 1.00 | 0.50 | 0.93 | 0.95 | 0.84 | 0.44 | 0.76 | 1.21 | 1.03 | 0.89 |
| Campy_If73 | 0.92 | 1.70 | 0.83 | 1.57 | 0.74 | 1.42 | 1.23 | 0.60 | 1.01 | 0.87 |
| Campy_If83 | 1.33 | 0.61 | 1.23 | 1.12 | 1.11 | 1.13 | 0.94 | 2.21 | 1.81 | 1.54 |
| Campy_If86 | 0.92 | 1.65 | 0.83 | 1.50 | 0.74 | 1.25 | 1.08 | 0.60 | 1.01 | 0.87 |
| Campy_If90 | 0.92 | 0.41 | 1.65 | 0.75 | 1.48 | 0.63 | 0.53 | 1.19 | 0.98 | 1.68 |
| Campy_IfM3 | 0.71 | 1.23 | 0.61 | 1.08 | 1.07 | 1.25 | 0.53 | 0.59 | 0.49 | 0.42 |
| Cj3 | 0.50 | 1.90 | 0.46 | 1.77 | 0.43 | 1.42 | 0.63 | 0.63 | 0.54 | 0.47 |
| Cj5 | 0.92 | 1.70 | 0.83 | 1.57 | 0.75 | 1.42 | 1.25 | 0.63 | 1.06 | 0.91 |
| Cj8 | 1.42 | 1.25 | 1.23 | 1.12 | 1.08 | 1.42 | 0.63 | 0.63 | 1.06 | 0.91 |
| Cj90 | 1.42 | 2.60 | 1.23 | 2.37 | 1.08 | 1.75 | 1.55 | 0.63 | 1.06 | 0.92 |
| Cj10 | 0.75 | 0.66 | 0.68 | 0.60 | 1.22 | 0.33 | 0.30 | 0.71 | 0.61 | 1.08 |
| Cj13 | 0.71 | 2.60 | 0.61 | 2.37 | 1.08 | 1.75 | 1.53 | 0.30 | 0.51 | 0.44 |
| Cj15 | 0.75 | 1.40 | 0.66 | 1.28 | 1.17 | 0.88 | 0.76 | 0.59 | 0.49 | 0.84 |
| Cj16 | 0.46 | 0.83 | 0.41 | 0.74 | 0.36 | 0.60 | 0.51 | 0.28 | 0.46 | 0.39 |
| Cj18 | 0.46 | 1.70 | 0.83 | 1.57 | 0.74 | 1.42 | 1.21 | 0.59 | 0.49 | 0.85 |
| Cj24 | 0.33 | 1.21 | 0.63 | 1.12 | 0.58 | 1.13 | 0.96 | 0.60 | 1.01 | 0.87 |
| ATCC 81-176 | 0.92 | 1.65 | 0.83 | 1.50 | 0.74 | 1.25 | 1.06 | 0.59 | 0.98 | 0.84 |
| BfR-CA-14430 | 0.46 | 0.81 | 0.41 | 0.73 | 0.37 | 1.17 | 0.49 | 0.59 | 0.49 | 0.42 |
| DSM 4688 | 0.71 | 1.25 | 0.63 | 1.13 | 1.10 | 0.75 | 0.66 | 0.68 | 0.57 | 0.49 |
| ***Campylobacter coli*** |  |  |  |  |  |  |  |  |  |  |
| Campy_If49 | 0.46 | 0.83 | 0.41 | 0.75 | 0.74 | 1.25 | 1.06 | 0.59 | 0.49 | 0.43 |
| Campy_If52 | 0.34 | 2.35 | 1.16 | 2.05 | 1.00 | 1.21 | 1.01 | 0.28 | 0.46 | 0.78 |
| Campy_If87 | 0.71 | 1.25 | 0.61 | 1.12 | 0.54 | 0.71 | 0.61 | 0.59 | 0.49 | 0.85 |
| Cc1 | 0.71 | 0.61 | 0.61 | 2.17 | 0.54 | 0.63 | 0.54 | 0.60 | 0.51 | 0.88 |
| Cc2 | 0.67 | 2.50 | 1.21 | 2.28 | 1.09 | 2.38 | 1.96 | 1.05 | 1.71 | 1.45 |
| Cc4 | 1.42 | 2.50 | 1.21 | 2.22 | 2.11 | 2.75 | 1.16 | 1.10 | 0.91 | 1.57 |
| Cc7 | 1.42 | 2.45 | 1.23 | 2.17 | 1.07 | 1.25 | 1.06 | 1.19 | 0.98 | 0.84 |
| Cc21 | 0.92 | 1.65 | 0.81 | 1.48 | 1.44 | 1.21 | 2.01 | 1.09 | 1.79 | 1.52 |
| BfR-CA-09557 | 0.46 | 0.85 | 0.41 | 0.78 | 0.73 | 1.38 | 0.59 | 0.56 | 0.95 | 0.83 |
| DSM 4689 | 0.46 | 0.83 | 0.81 | 1.48 | 0.72 | 1.21 | 0.51 | 0.55 | 0.46 | 0.39 |
